# The Reliability of a Novel Automated System for ANA Immunofluorescence Analysis in Daily Clinical Practice

**DOI:** 10.1155/2016/6019268

**Published:** 2016-05-09

**Authors:** Mohammed Alsuwaidi, Margit Dollinger, Martin Fleck, Boris Ehrenstein

**Affiliations:** ^1^Department of Rheumatology and Clinical Immunology, Asklepios Medical Center Bad Abbach, Kaiser-Karl-V-Allee 3, 93077 Bad Abbach, Germany; ^2^Department of Internal Medicine I, University Medical Center Regensburg, 93042 Regensburg, Germany

## Abstract

Automated interpretation (AI) systems for antinuclear antibody (ANA) analysis have been introduced based on assessment of indirect immunofluorescence (IIF) patterns. The diagnostic performance of a novel automated IIF reading system was compared with visual interpretation (VI) of IIF in daily clinical practice to evaluate the reduction of workload. ANA-IIF tests of consecutive serum samples from patients with suspected connective tissue disease were carried out using HEp-2 cells according to routine clinical care. AI was performed using a visual analyser (Zenit G-Sight, Menarini, Germany). Agreement rates between ANA results by AI and VI were calculated. Of the 336 samples investigated, VI yielded 205 (61%) negative, 42 (13%) ambiguous, and 89 (26%) positive results, whereas 82 (24%) were determined to be negative, 176 (52%) ambiguous, and 78 (24%) positive by AI. AI displayed a diagnostic accuracy of 175/336 samples (52%) with a kappa coefficient of 0.34 compared to VI being the gold standard. Solely relying on AI, with VI only performed for all ambiguous samples by AI, would have missed 1 of 89 (1%) positive results by VI and misclassified 2 of 205 (1%) negative results by VI as positive. The use of AI in daily clinical practice resulted only in a moderate reduction of the VI workload (82 of 336 samples: 24%).

## 1. Introduction

The screening for antinuclear antibodies (ANA) utilizing indirect immunofluorescence (IIF) on human epithelial-2 (HEp-2) cells has been established as the standard method to detect the presence of ANA in sera of patients suffering from various autoimmune conditions including connective tissue diseases [1–6]. This approach was recommended as the preferred method for ANA screening by the American College of Rheumatology and allows for the detection of autoantibodies directed towards more than 100 different nuclear or cytoplasmic autoantigens resulting in different IIF patterns [5, 7]. Therefore, laboratories performing ANA-IIF tests should report the ANA titre as well as the recognized pattern [1, 5]. However, there is no standardization for ANA-IIF testing established, and this method is time-consuming, labour-intensive, and prone to an investigator related bias due to the subjectivity of visual interpretation (VI) [1]. Therefore, strong efforts have been made to develop automated interpretation (AI) systems for ANA analysis based on assessment of IIF patterns. Currently, there are at least six different systems available that rely on the automated digital acquisition of IIF images combined with algorithms for subsequent discrimination of positive and negative results [7, 8]. In addition, classification of positive IIF patterns is provided by some of these systems [8]. However, studies analysing the reliability of these systems in routine clinical practice are limited, and the potential to reduce the workload has not been determined so far [7]. Therefore, the diagnostic performance of a novel automated IIF reading system was compared with VI of IIF in daily clinical practice. Furthermore, the impact of AI on the workload was calculated.

## 2. Methods

### 2.1. Serum Samples

The study was conducted in the immunology laboratory of a tertiary care medical center attached to the University of Regensburg specializing in the treatment of autoimmune mediated rheumatic diseases. All serum samples submitted for ANA-IIF testing from routine clinical care over the period of one month were included. The study was conducted according to the regulations for laboratory validation studies set by the local institutional review board.

### 2.2. Indirect Immunofluorescence Staining

The ANA-IIF testing of serum samples for routine clinical care was performed using an assay with slides coated with HEp-2 cells (further referred to as “ANA-IIF-assay 1”; IFA ANA HEp-2 IgG, Lot AHEPo 0102-12, Viro-Immun, Germany). Results for ANA-IIF-assay 1 were obtained by visual interpretation of the fluorescence patterns observed using a LED fluorescence microscope (imLD, BioSystems, Germany).

In parallel, all samples were also subjected to a second ANA-IIF assay utilizing slides coated with HEp-2 cells (further referred to as “ANA-IIF-assay 2”; ImmunoConcepts Fluorescent HEp-2 ANA, Lot 1110014, A. Menarini Diagnostics, Germany). The results of the ANA-IIF-assay 2 were interpreted visually utilizing the fluorescence microscope (imLD, BioSystems, Germany) and were also subjected to image acquisition and interpretation by an automated LED fluorescence microscope scanner (Zenit G-Sight, A. Menarini Diagnostics, Germany). The software provided by the manufacturer automatically interpreted the generated fluorescence images (version 1.06.00, A. Menarini Diagnostics, Germany).

In general, serum samples were initially evaluated in a dilution of 1 : 80. If a positive result was obtained either by visual or automated interpretation, further dilutions (1 : 160, 1 : 320, 1 : 640, 1 : 1280, 1 : 2560, 1 : 5120, 1 : 10240, and 1 : 20480) were conducted in a stepwise manner (2-3 dilutions for each step) until either a negative result or a positive result for the highest dilution (1 : 20480) was obtained. For a small subset of samples, originating from patients, who already had a positive ANA-IIF-assay 1 result prior to the current study at our institution, the initial ANA-IIF evaluation was performed for the titre and adjoining titres of the previous test result.

### 2.3. Visual Interpretation

Two technicians (examiner 1 and examiner 2) with longstanding experience in the interpretation of ANA-IIF results independently rated ANA-IIF of both assays visually using the manual operated LED fluorescence microscope. ANA-IIF tests with no detectable IIF were rated negative, tests with a defined staining pattern were rated positive, and tests with detectable fluorescence albeit no clearly detectable staining pattern were rated ambiguous. Positive ANA-IIF results were categorized by the predominant fluorescence pattern in the highest dilution with a positive result as being either homogeneous, fine speckled, coarse speckled, nucleolar, centromeric, mitochondrial, or nuclear dots.

To define a gold standard for results of the ANA-IIF-assay 2, visual interpretation of the digitized images generated by the automated reader was performed by both examiners in conjunction with a third experienced examiner.

### 2.4. Automated Interpretation

Automated analyses of the ANA-IIF-assay 2 images were obtained using the following cut-off values for the fluorescence index calculated by the software for each image: for an index < 15 as negative, for an index between 15 and 25 as ambiguous, and for an index > 25 as positive. With a separate algorithm, the software categorized ANA-IIF patterns as being either negative, homogeneous, fine speckled, coarse speckled, nucleolar, centromeric, mitochondrial, or nuclear dots. With regard to samples with a positive ANA-IIF-assay 2 result, the highest dilution titre still revealing a positive result and the corresponding ANA-IIF pattern were both recorded for further analyses.

### 2.5. Antibodies against Extractable Nuclear Antigens and Double Stranded DNA

The analyses of antibodies against extractable nuclear antigens (ENA) and double stranded DNA (dsDNA) were not performed as part of the current research protocol, but we retrieved these results, if performed in routine clinical care. Enzyme-linked immunosorbent assay (ELISA) tests for the detection of antibodies against ENA or dsDNA were either ordered by the treating physicians or performed according to the standard operating procedures of the laboratory for further evaluation of samples with a positive or ambiguous ANA-IIF-assay 1 result. Samples with a homogeneous ANA-IIF pattern were tested for antibodies against dsDNA and samples with speckled, centromeric, nucleolar, or nuclear dots patterns for antibodies against ENA on a commercially available automated ELISA platform (detection of antibodies against ENA (SM, RNP/SM, SSA, SSB, Scl-70, Jo-1, CentrB, and RNP-70) and against dsDNA, Alegria*™* ANA-Screen and Alegria anti-dsDNA IgG, Orgentec, Germany).

### 2.6. Statistical Analysis

Data were entered and analysed using Microsoft Excel software. To demonstrate the differences between the visual and automated analyses of ANA-IIF-assay 2, crosstabulations for result categories (negative, ambiguous, and positive) for all samples and ANA-IIF titres and patterns for the subset with a positive result by the visual gold standard were performed. As a secondary analysis, we dichotomized the former analyses accounting negative as well as ambiguous results as negative and positive results as positive.

Additionally, we determined the concordance, sensitivity, specificity, and unweighted kappa statistic (SPSS, version 19, IBM, USA) for comparisons between the gold standard and the individual visual interpretation of both examiners for both ANA-IIF assays and the automated interpretation of ANA-IIF-assay 2.

## 3. Results

### 3.1. Automated versus Visual Interpretation of ANA Indirect Immunofluorescence

Of the 336 samples investigated, the gold standard visual interpretation (ANA-IIF-assay 2 rated by three examiners) yielded 205 (61%) negative, 42 (13%) ambiguous, and 89 (26%) positive results, whereas 82 (24%) were determined to be negative, 176 (52%) ambiguous, and 78 (24%) positive by automated interpretation (Table 1).

Of the 82 negative samples by the automated analyses, 78 (95%) were also negative, 3 (4%) were ambiguous, and 1 (1%) was positive as judged by the gold standard. Of the 176 ambiguous samples by automated analyses, 125 (71%) were negative, 30 (17%) ambiguous, and 21 (12%) positive by the gold standard visual analysis. Of the 78 positive samples by automated analyses, 2 (3%) were negative by visual analysis, 9 (12%) ambiguous, and 67 (86%) also positive. Automatic analysis displayed a diagnostic accuracy of 175/336 samples (52%) with a kappa coefficient of 0.34 compared to visual interpretation as the gold standard. Solely relying on automated interpretation with visual interpretation only performed for all ambiguous samples by automated interpretation would have missed 1 of 89 (1%) positive results by visual interpretation and misclassified 2 of 205 (1%) negative results as positive.

When ambiguous results were classified as being negative, of the 258 negative samples by the automated interpretation, 236 (91%) were also negative and 22 (9%) were positive as judged by the gold standard visual interpretation, revealing a concordance of 303/336 (90%), a sensitivity of 67/89 (75%), and a specificity of 236/258 (91%), with a kappa coefficient of 0.74.

### 3.2. Automated versus Visual Interpretation of ANA Indirect Immunofluorescence Titres and Patterns

Of the 89 positive samples by the gold standard visual interpretation of ANA-IIF-assay 2, automated interpretation correctly identified 54/89 (61%) ANA-IIF titres with a corresponding kappa coefficient of 0.55 (Tables 2 and 4). Automated interpretation identified 35/89 (39%) ANA-IIF patterns correctly (Tables 3 and 4): 18 (95%) of 19 homogeneous, 1 (3%) of 36 fine speckled, 13 (65%) of 20 coarse speckled, 1 (11%) of 9 nucleolar, 1 (33%) of 3 centromeric, 1 (100%) of 1 mitochondrial, and 0 (0%) of 1 nuclear dots patterns, with a corresponding kappa coefficient of 0.28. Of the 176 results rated by automated interpretation as ambiguous, 5 samples (3%) were shown by the software to display a specific pattern (1 homogeneous, 1 coarse speckled, 2 nucleolar, and 1 centromeric).

The diagnostic performance of the automated compared to the visual analyses by two individual examiners of ANA-IIF for all 336 serum samples and the subset of 89 samples, which revealed a positive result by visual interpretation of the ANA-IIF-assay 2, is outlined in Table 4.

### 3.3. Antibodies against Extractable Nuclear Antigens and Double Stranded DNA

Of the 336 samples, 168 (50%) were tested for antibodies against extractable nuclear antigens (ENA) and 166 (49%) for antibodies against double stranded DNA (dsDNA) and 159 (47%) for both during routine clinical care.

Of the 89 positive samples by the gold standard visual interpretation of the ANA-IIF-assay 2, 26/81 (32%) of tested samples were positive for antibodies against ENA and 18/79 (23%) against dsDNA, of the 42 ambiguous samples 1/30 (3%) was positive for antibodies against ENA and 2/27 (7%) were positive for antibodies against dsDNA, and of the 205 negative samples 2/57 (2%) were positive for antibodies against ENA and 5/60 (8%) against dsDNA.

Of the 22 samples that were negative or ambiguous by automated and positive by the gold standard visual interpretation, 4/19 (21%) were positive for antibodies against ENA (*n* = 1 anti-Jo-1 > 200 U/mL (normal < 25), *n* = 2 anti-SSA 111 and 187 U/mL (normal < 25) and *n* = 1 anti-CentrB 128 U/mL (normal < 10 U/mL)) and 2/10 (10%) against dsDNA (23 and 36 IU/mL (normal < 20)).

## 4. Discussion

Automated acquisition of images and computer-aided analysis of patterns have been introduced to facilitate and standardize ANA-IIF testing. However, many of the studies evaluating AI for ANA analysis utilized well-characterized serum samples producing distinct IIF patterns [8–11]. In addition, the impact of ANA-AI on the workload in a routine laboratory was not addressed in the majority of these previous studies [8–14]. Therefore, this study analysed the diagnostic performance of a novel automated IIF reading system in comparison with VI of IIF in daily clinical practice.

Recently, a comparison of six different AI systems in the IIF analysis of the same 144 serum samples demonstrated a good screening consistency for all systems with a total sensitivity rate of 96.7% and specificity rate of 89.9% [8]. Our findings are in line with this study as well as other reports on the performance of our and similar automated interpretation systems [9, 10, 12–16] as the automated interpretation system used in our study provided a reliable discrimination between positive and negative results. Utilizing novel algorithms, AI of ANA-IIF demonstrated a diagnostic accuracy of 52% with a kappa coefficient of 0.34 compared to VI as the gold standard. If all ambiguous results were classified as being negative, the concordance increased even to 90%, with a sensitivity of 75% and a specificity of 91%, corresponding to a kappa coefficient of 0.74. These results highlight the strength of AI as a potential screening tool for identification of positive IIF tests, which could ultimately result in a substantial reduction of the workload in routine diagnostic evaluations.

However, in contrast to previous studies utilizing well-defined biobank samples [8, 10, 13], the use of AI in daily clinical practice in our study resulted only in a moderate reduction of the VI workload (82 of 336 samples: 24%), which was predominantly due to a large proportion of ambiguous AI results. Due to the mode of data collection of our study (recording AI results only in categories and not the fluorescence index), further sensitivity analyses for different cut-off values, other than those recommended by the manufacturer, could not be performed.

Usually, ambiguous IIF findings are not adequately represented in biobank samples, and therefore the effectiveness of AI might have been overinterpreted in previous studies relying on the analysis of sera containing ANA at high titres and well-defined IIF patterns. In addition, the reliability of screening assays is strongly related to the pretest probability [7]. Therefore, the number of ambiguous and positive IIF-test results will be considerably higher in facilities analysing serum samples obtained from specialized rheumatology centers in comparison to laboratories obtaining nonspecialized referrals.

Description of the IIF pattern is an essential piece of information, which should be reported on together with the titre for each test performed as recommended by the ACR [5]. Despite utilizing novel algorithms in our AI system, we observed remarkable limitations in IIF pattern recognition. This finding has been reported for all of the tested AI systems so far and reflects the challenging task to identify distinct IIF patterns reliably in an automated fashion [8]. The accuracy of AI will be even more affected in the simultaneous presence of multiple ANA specificities in the same patient, which can be observed in a substantial number of patients suffering from connective tissue diseases.

## 5. Conclusion

In view of these results, we propose a two-step approach for ANA evaluation with an ANA screening based on AI as first step and confirmation as well as pattern analysis of ambiguous and positive findings by VI as a second step. However, due to the limited reduction of the workload for laboratories obtaining sera from patients with a high pretest probability of a positive or ambiguous IIF result, this approach might be suitable particularly for high throughput laboratories with a limited number of specialized referrals.

## Figures and Tables

**Table 1:** Visual versus automated detection of antinuclear antibodies by indirect immunofluorescence. Crosstabulation of the results of the visual (gold standard) versus automated interpretation of antinuclear antibodies (ANA) detected by indirect immunofluorescence (IIF) for 336 serum samples utilizing the ANA-IIF-assay 2.

Visual interpretation (gold standard)	Automated interpretation, *n* (%)			
	Negative	Ambiguous	Positive	Total
Negative	**78** (95)	125 (71)	2 (3)	205 (61)
Ambiguous	3 (4)	**30** (17)	9 (12)	42 (13)
Positive	1 (1)	21 (12)	**67** (86)	89 (26)

Total	82 (100)	176 (100)	78 (100)	336 (100)

**Table 2:** Visual versus automated detected titres of antinuclear antibodies by indirect immunofluorescence. Crosstabulation of antinuclear antibodies (ANA) titres by indirect immunofluorescence (IIF) for 89 serum samples that revealed a positive result by visual interpretation of the ANA-IIF-assay 2; concordant results are shown in bold (NEG: negative; AMB: ambiguous).

Visual interpretation (gold standard)	Automated interpretation, *n*											
	NEG	AMB	1 : 80	1 : 160	1 : 320	1 : 640	1 : 1280	1 : 2560	1 : 5120	1 : 10240	1 : 20480	Total
1 : 80	1	15	**16**	1								**33**
1 : 160		2	2	**10**	1							**15**
1 : 320		1	1	1	**6**							**9**
1 : 640		3	1	1	1	**5**						**11**
1 : 1280					1		**6**	1				**8**
1 : 2560								**7**				**7**
1 : 5120								1	**2**			**3**
1 : 10240										**1**		**1**
1 : 20480										1	**1**	**2**

Total	**1**	**21**	**20**	**13**	**9**	**5**	**6**	**9**	**2**	**2**	**1**	**89**

**Table 3:** Visual versus automated detected patterns of antinuclear antibodies by indirect immunofluorescence. Crosstabulation of antinuclear antibodies (ANA) titres by indirect immunofluorescence (IIF) for 89 serum samples that revealed a positive result by visual interpretation of the ANA-IIF-assay 2; concordant results are shown in bold.

Visual interpretation (gold standard)	Automated interpretation, *n*							
	Negative	Homogeneous	Fine speckled	Coarse speckled	Nucleolar	Centromeric	Mitochondrial	Total
Homogeneous	1	**18**						**19**
Fine speckled	7	11	**1**	13		3	1	**36**
Coarse speckled	4	1		**13**	1	1		**20**
Nucleolar	4			3	**1**	1		**9**
Centromeric	1			1		**1**		**3**
Mitochondrial							**1**	**1**
Nuclear dots	1							**1**

Total	**18**	**30**	**1**	**30**	**2**	**6**	**2**	**89**

**Table 4:** Diagnostic performance of automated indirect immunofluorescence ANA detection in comparison to visually obtained results by 2 individual examiners utilizing 2 different assays. Crosstabulation of the diagnostic performance of the automated versus visual analyses by individual examiners of antinuclear antibodies (ANA) by indirect immunofluorescence (IIF) for all 336 serum samples and the subset of 89 that revealed a positive result by visual interpretation of the ANA-IIF-assay 2 (NEG: negative; AMB: ambiguous; POS: positive).

Comparison to the gold standard, derived by visual evaluation of assay 2 by 3 examiners	Automated analyses	Visually by examiner 1	Visually by examiner 2	Visually by examiner 1	Visually by examiner 2
	Assay 2	Assay 2	Assay 2	Assay 1	Assay 1
*By category (n* = 336): *NEG, AMB, or POS*					
Concordance (%)	52	86	86	80	78
Kappa	0.34	0.75	0.75	0.59	0.55

*By category (n* = 336*): NEG or POS (AMB = NEG)*					
Concordance (%)	90	93	94	91	90
Sensitivity (%)	75	87	89	70	65
Specificity (%)	96	96	96	99	99
Kappa	0.74	0.83	0.85	0.75	0.71

*By titre (n* = 89)					
Concordance (%)	61	69	72	27	21
Kappa	0.55	0.63	0.66	0.15	0.10

*By pattern (n* = 89)					
Concordance (%)	36	76	69	47	46
Kappa	0.28	0.67	0.52	0.33	0.32
